# 1466. Antiretroviral (ART) Virologic Suppression (VS) and Patient Reported Outcomes (PROs) at 6 Months in the Clinical Opportunities and Management to Exploit Bictegravir/Emtricitabine/Tenofovir Alafenamide (B/F/TAF) an Asynchronous Connection Key (COMEBACK) Study

**DOI:** 10.1093/ofid/ofac492.1293

**Published:** 2022-12-15

**Authors:** Lindsey Roden, Gregory Huhn, Camille E DeMarco, Kody Keckler, Patricia Cortes Valadez, Kerianne Burke

**Affiliations:** The Ruth M. Rothstein CORE Center, Chicago, Illinois; The Ruth M. Rothstein CORE CENTER, Chicago, Illinois; The Ruth M. Rothstein CORE Center, Chicago, Illinois; The Ruth M. Rothstein CORE Center, Chicago, Illinois; The Ruth M. Rothstein CORE Center, Chicago, Illinois; The Ruth M. Rothstein CORE Center, Chicago, Illinois

## Abstract

**Background:**

Effectively interrupting the source of transmission is a critical step in ending the HIV epidemic. COMEBACK (NCT04519970) is a 48-week single-center study in Chicago implemented in September 2020, with its main objectives to reengage lost-to-care patients and rapidly reinitiate ART to promote VS and favorable PROs.

**Methods:**

Adults off ART ≥2 weeks, without history of significant B/F/TAF resistance or renal impairment, were rapidly started on B/F/TAF upon reengagement after same day collection of baseline labs and PROs. A retention screening assessment was used to stratify participants into case management (CM) tiers: Minimal, Moderate, or Advanced. An acuity assessment tool was adapted to determine whether participants needed additional support based on retention and VS. Currently, 80 of the expected 100 subjects are enrolled and 55 have reached the 24-week timepoint. Baseline and 6-month endpoints were analyzed for these participants.

**Results:**

At baseline (N=55), median age was 34 years (range, 24–62), with 92.6% Black and 72.2% cisgender male. Median CD4+ was 338 cells/mm^3^, with a median viral load 7,402copies/mL, (range, < 40–333,350, 16.3% VS). Median time off ART was 2.6 months (range, 0.5-243). For CM, participants were stratified into Minimal (71%) and Moderate (29%) tiers; none were identified as Advanced.

Table 1 reflects tier shifts through 24 weeks.

Shifts in CM intensity differs from the HIV adherence self-efficacy PRO completed within 24 weeks, indicating that at least 50% underestimated their need to integrate and maintain adherence to ART treatment. Forty of 55 participants (72.7%) were retained-in-care at 6 months, with VS in 61.8% (N=34/55) by intention-to-treat and 85% (N=34/40) by observed analysis. No resistance to B/F/TAF was detected through 6 months.

**Figure:**
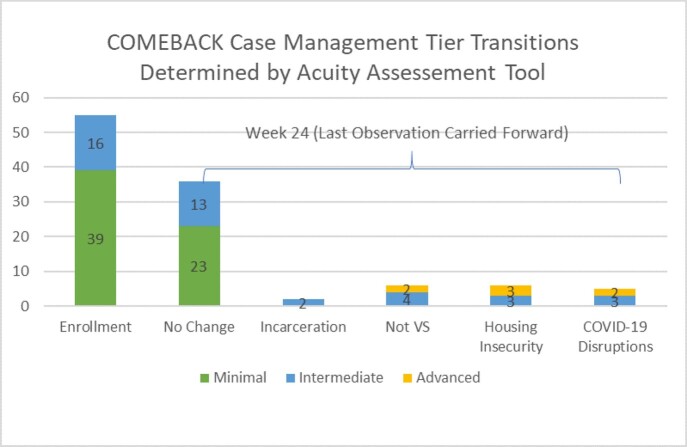

Note: The table reflects patients retained on study at their week 24 endpoint.

**Conclusion:**

VS was high for participants retained-in-care, but lapses in retention and shifts toward more intense CM were likely due to social determinants of health challenges, including incarceration, housing insecurity, and COVID-19-related disruptions in healthcare.

**Disclosures:**

**Gregory Huhn, MD, MPHTM**, Eli Lilly: Advisor/Consultant|Eli Lilly: Grant/Research Support|Gilead: Advisor/Consultant|Gilead: Grant/Research Support|Jannsen: Advisor/Consultant|Jannsen: Grant/Research Support|Merck: Advisor/Consultant|Viiv: Advisor/Consultant|Viiv: Grant/Research Support.

